# Impact of 6-month triptorelin formulation on predicted adult height and basal gonadotropin levels in patients with central precocious puberty

**DOI:** 10.3389/fendo.2023.1134977

**Published:** 2023-02-17

**Authors:** Eunjoo Yoo, Sinae Kim, Hye Lim Jung, Jung Yeon Shim, Jae Won Shim, Deok Soo Kim, Ji Hee Kwak, Eun Sil Kim, Aram Yang

**Affiliations:** ^1^ Department of Pediatrics, Kangbuk Samsung Hospital, School of Medicine, Sungkyunkwan University, Seoul, Republic of Korea; ^2^ Biostatistics Collaboration Team, Research Core Center, National Cancer Center, Goyang, Republic of Korea

**Keywords:** central precocious puberty, gonadotrophin-releasing hormone analogue, gonadotropins, triptorelin pamoate, 6-month formulation

## Abstract

**Background:**

Triptorelin, a long-acting gonadotropin-releasing hormone (GnRH) agonist, is available in 1-, 3-, and 6-month formulations to treat central precocious puberty (CPP). The triptorelin pamoate 22.5-mg 6-month formulation recently approved for CPP offers greater convenience to children by reducing the injection frequency. However, worldwide research on using the 6-month formulation to treat CPP is scarce. This study aimed to determine the impact of the 6-month formulation on predicted adult height (PAH), changes in gonadotropin levels, and related variables.

**Methods:**

We included 42 patients (33 girls and nine boys) with idiopathic CPP treated with a 6-month triptorelin (6-mo TP) formulation for over 12 months. Auxological parameters, including chronological age, bone age, height (cm and standard deviation score [SDS]), weight (kg and SDS), target height (TH), and Tanner stage, were evaluated at baseline, and after 6, 12, and 18 months of treatment. Hormonal parameters, including serum luteinizing hormone (LH), follicle-stimulating hormone (FSH), and estradiol for girls or testosterone for boys, were analyzed concurrently.

**Results:**

The mean age at treatment initiation was 8.6 ± 0.83 (8.3 ± 0.62 for girls, 9.6 ± 0.68 for boys). The peak LH level following intravenous GnRH stimulation at diagnosis was 15.47 ± 9.94 IU/L. No progression of the modified Tanner stage was observed during treatment. Compared to baseline, LH, FSH, estradiol, and testosterone were significantly reduced. In particular, the basal LH levels were well suppressed to less than l.0 IU/L, and the LH/FSH ratio was less than 0.66. The bone age/chronological age ratio remained stable with a decreasing trend (1.15 at the start of treatment, 1.13 at 12 months, 1.11 at 18 months). PAH SDS increased during treatment (0.77 ± 0.79 at baseline, 0.87 ± 0.84 at the start of treatment, 1.01 ± 0.93 at six months, and 0.91 ± 0.79 at 12 months). No adverse effects were observed during treatment.

**Conclusion:**

The 6-mo TP suppressed the pituitary-gonadal axis stably and improved the PAH during treatment. Considering its convenience and effectiveness, a significant shift to long-acting formulations can be expected.

## 1 Introduction

Central precocious puberty (CPP) is defined as the onset of pubertal development—breast development before the age of eight in girls, or testicular development to over 4 mL before the age of nine in boys, due to premature activation of the hypothalamic-pituitary-gonadal axis (1). It occurs 5- to 10-fold more frequently in girls than in boys, and is usually sporadic. The cause is idiopathic in approximately 90% of the girls, while a structural central nervous system abnormality may occur in 26–75% of the boys with CPP (2, 3). Early pubertal changes lead to accelerated growth, bone maturation, and tall stature during childhood, often resulting in reduced adult height due to premature growth plate fusion (4). Moreover, the high sex hormone levels in girls with CPP cause early menarche, which might lead to psychosocial problems (5). However, when the loss of predicted adult height (PAH) or social problems due to early sexual development are unlikely to be significant, the need for treatment should be evaluated through periodic observation every 3–6 months (6–8).

Since the mid-1980s, long-acting gonadotropin-releasing hormone (GnRH) agonists have been the gold standard treatment for CPP. GnRH agonists stimulate the pituitary gonadotrophs continuously, leading to desensitization and decreased release of luteinizing hormone (LH) and follicle-stimulating hormone (FSH) (9). Depot or slow-release GnRH agonist formulations have been developed, significantly prolonging the drug action and improving patient compliance. The demand for these drugs has been increasing, especially during the COVID-19 pandemic (10). Triptorelin, one of these drugs, is available in 1-month (3.75 mg), 3-month (11.25 mg), or 6-month (22.5 mg) formulations (1, 11, 12).

The prevalence of CPP has been increasing worldwide (13), particularly in Korea (14, 15). Six-month triptorelin pamoate (6-mo TP) was approved for CPP treatment in Korea in March 2020. However, since only a few large hospitals can prescribe it, the available data about its efficacy and safety are scarce (12, 16).

This study aimed to determine the impact of the 6-mo TP formulation on the PAH, changes in gonadotropin levels, and related variables.

## 2 Materials and methods

### 2.1 Study design and patients

The data of all patients diagnosed with CPP based on the International Classification of Diseases 10th Revision (ICD-10; code E22.8) between January 2019 and March 2022 at a tertiary university hospital in Seoul, Republic of Korea, were retrospectively reviewed. Only those who met all the inclusion criteria were included in this study.

The inclusion criteria were based on a consensus statement (1): (1) objective breast enlargement before the age of eight in girls or testicular volume greater than 4 mL before the age of nine in boys; (2) bone age over a year ahead of the chronological age; (3) pubertal LH response to a GnRH stimulation test; (4) intramuscularly administrated 6-mo TP 31 mg (22.5 mg as triptorelin; Diphereline SR 22.5 mg Inj., Ipsen, France) for at least two doses. Patients treated with the 6-mo TP formulation for less than a year or who had other organic causes for early pubertal development (obstetrical problem, brain tumor, congenital adrenal hyperplasia, hypothyroidism, or a history of cranial irradiation) or were receiving recombinant growth hormone therapy were excluded. Girls with ovarian disease diagnosed by transabdominal ultrasonography and boys diagnosed with organic brain problems by sellar magnetic resonance imaging were excluded (16).

GnRH stimulation tests were performed at diagnosis by intravenous (IV) administration of 100 μg fixed-dose Gonadorelin (Relefact; Sanofi-Aventis, Frankfurt am Main, Germany). Serum LH and FSH levels were assessed before and 30, 45, 60, and 90 minutes after stimulation using an immunoradiometric assay (BioSource SA, Nivelles, Belgium). A peak LH level above 5 mIU/mL was considered diagnostic for CPP (17, 18).

The Institutional Review Board of the Kangbuk Samsung Hospital approved this study (IRB number KBSMC 2022-12-050) and waived the requirement for informed consent owing to the retrospective design of the study. Besides, the patients’ information was anonymized and de-identified before analysis.

### 2.2 Assessments

The primary outcome measure was basal LH suppression to prepubertal level. Secondary outcome measures were suppression of sex hormones, slowing the progression in the development of secondary sexual characteristics and bone maturation and increasing PAH (19).

Blood samples for random serum LH, FSH, and estradiol for girls or testosterone for boys were collected pre-injection every six months during treatment to assess gonadotropin suppression. LH and FSH levels without stimulation at diagnosis and each visit were referred to as basal LH and FSH. Effective LH suppression to prepubertal levels was defined as basal LH <1 mIU/mL (17, 20–22). Prepubertal basal FSH was defined as <2.5 mIU/mL (23), and basal LH/FSH ratio as <0.66 (24, 25). The lower limits of LH and FSH detection were 0.2 mIU/mL and 0.7 mIU/mL, respectively. Estradiol and testosterone reference values vary with the assessment method and assay used (1, 3, 5). Based on the laboratory cutoff values used in this study, estradiol above 12.3 pg/mL and testosterone above 0.481 ng/mL were considered pubertal levels (26).

At the time of CPP diagnosis, and every six months from 6-mo TP administration, the patients visited the outpatient clinic to measure height and weight, and the body mass index (BMI) was calculated. Standard deviation scores (SDS) of height, weight, and BMI were calculated according to the 2017 Korean National Growth Charts for children and adolescents (27). Bone age was measured by an experienced pediatric endocrinologist and radiologist using left wrist radiographs and following the Greulich-Pyle Method (28). The bone age/chronological age ratio was used as an index of bone age advancement (3). Sexual development was assessed using Tanner staging at each outpatient visit. Testicular volume was measured by an experienced pediatric endocrinologist using an orchidometer. Breast examination was performed through breast inspection and palpation according to the modified Tanner method (3). Midparenal height was set as the patient’s target height (TH) and calculated as follows: (father’s height + mother’s height)/2 ± 6.5 cm (–6.5 cm for girls and +6.5 cm for boys) (29). PAH was calculated using the Bayley-Pinneau average age method (30). Adverse events were monitored during the treatment period (31).

### 2.3 Statistical analysis

Data were analyzed using IBM SPSS Statistics, Version 24.0 (IBM Corp., Armonk, NY, USA). Averages over time of hormonal parameters, bone age/chronological age ratio, and PAH SDS were compared by a linear mixed model, and an overall *p*-value < 0.05 compared at the time of diagnosis was considered statistically significant. *Post-hoc* analysis and Bonferroni corrected *p* values were used to compare parameters between time points and at diagnosis.

## 3 Results

### 3.1 Patient characteristics

This study included 42 patients (33 females and nine males) with idiopathic CPP. The clinical, demographic, and laboratory baseline characteristics of the patients are shown in **Table 1**. The age at GnRH agonist treatment initiation was 8.33 ± 0.62 for girls and 9.65 ± 0.68 for boys. The starting dose was administrated as a 1- or 3-month depot TP and changed to 6-mo TP at a mean age of 8.80 ± 0.70 for girls and 9.88 ± 0.63 for boys. Changes in the formulation were made to reduce the frequency of pain resulting from the injection, increase convenience, and improve compliance. The 6-mo formulation change occurred at an average of 4 months after treatment initiation after three injections of the 1-month dosage form or one injection of the 3-month dosage form. The 6-mo TP was prescribed and administered four times in 16 patients, three times in 14, and twice in 12, with an average of 3.10 ± 0.80 times. The peak LH level following IV GnRH stimulation was 15.47 ± 9.94 IU/L at diagnosis of CPP.

**Table 1:** Clinical, demographic, and laboratory characteristics of the patients at baseline.

Variable	Total (*n* = 42)		Girls (*n* = 33)	Boys (*n* = 9)		
Age at diagnosis (years)	8.61 ± 0.83		8.33 ± 0.62	9.65 ± 0.68		
Age at start of 6-month formulation (years)	9.02 ± 0.81		8.80 ± 0.70	9.88 ± 0.63		
BA at diagnosis (years)	9.54 ± 1.11		9.27 ± 11.19	10.54 ± 14.05		
BA/CA ratio at diagnosis	1.11 ± 0.09		1.11 ± 0.1	1.09 ± 0.08	
Weight (kg)	32.41 ± 8.48		29.12 ± 4.04	44.47 ± 9.75		
Height (cm)	135.36 ± 7.12		132.73 ± 4.74	145 ± 6.09		
BMI (kg/m^2^)	17.45 ± 2.89		16.48 ± 1.54	21.03 ± 3.86		
Weight (SDS)	0.47 ± 1.18		0.29 ± 0.85	1.13 ± 1.89		
Height (SDS)	0.80 ± 0.90		0.66 ± 0.85	1.31 ± 0.96		
BMI (SDS)	0.54 ± 1.02		0.28 ± 0.69	1.47 ± 1.46		
TH (cm)	164.74 ± 6.11		162.21 ± 3.57	174 ± 4.19		
TH (SDS)	0.24 ± 0.7		0.28 ± 0.69	0.11 ± 0.74		
Tanner stage ^†^	2.26 ± 0.59		2.27 ± 0.52	2.22 ± 0.83		
Peak LH at diagnosis (mIU/L)	15.47 ± 9.94		14.15 ± 9.43	20.30 ± 10.84		

All values are presented as means ± standard deviation. BA, bone age; CA, chronological age; BMI, body mass index; TH, target height; SDS, standard deviation score.

^†^breast development scale for girls, external genitalia scale for boys using testicular volume.

### 3.2 Changes in the hormonal parameters

The basal LH level at diagnosis, 0.72 ± 0.86 mIU/mL, declined to 0.58 ± 0.69 mIU/mL by the time of formulation change. Its levels 6, 12, and 18 months after the formulation change were 0.43 ± 0.16, 0.43 ± 0.16, and 0.46 ± 0.12 mIU/mL, respectively. Basal LH level at follow-ups were lower than that at the time of diagnosis. However, this difference was not significant (overall *p* =0.109)

**Figure 1:**
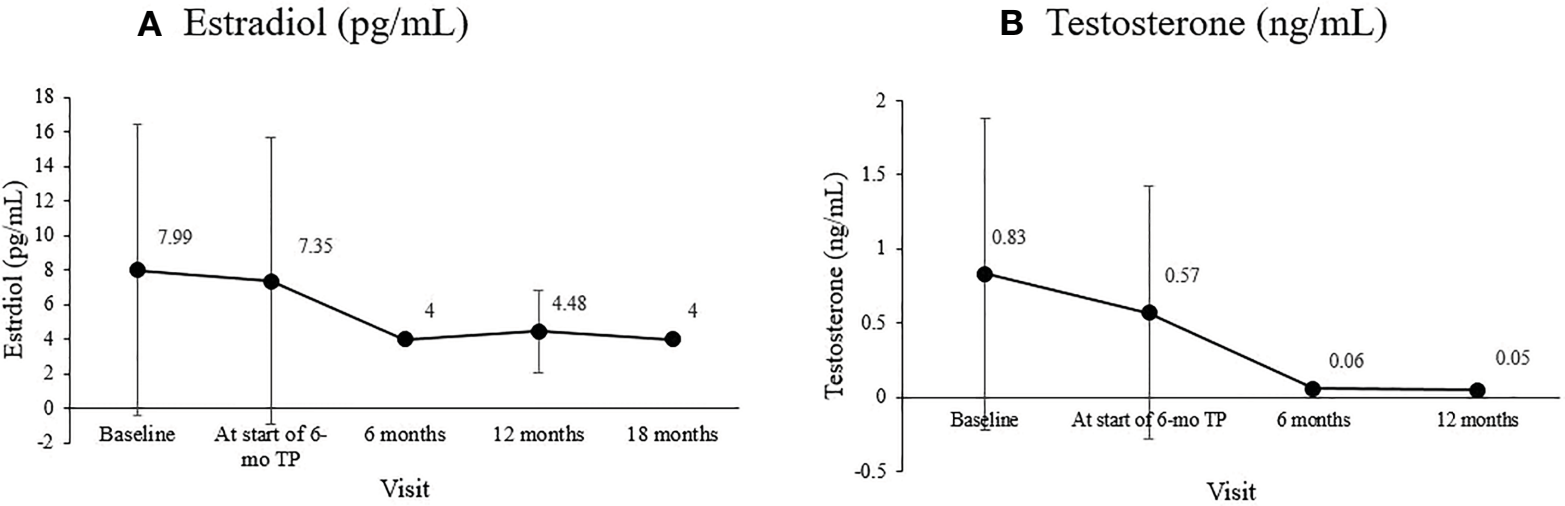
Changes in the mean sex hormone levels during treatment. **(A)** Estradiol (pg/mL; overall P =0.006). **(B)** Testosterone (ng/mL; overall p = 0.040).

The basal FSH level at the start of the 6-mo TP treatment was 2.52 ± 1.76 mIU/mL, and it was 2.06 ± 3.58, 1.74 ± 1.00, and 2. 15 ± 0.82 mIU/mL 6, 12, and 18 months later (overall *p* = 0.049). The LH/FSH ratio remained well below 0.4 throughout treatment without any significant change (overall *p* = 0.098). The sex hormones significantly decreased in both girls and boys (**Figures 1A, B**). In girls, estradiol decreased significantly from the level at the formulation change time (7.35 ± 8.28 pg/mL) to 4.00 ± 0.00, 4.48 ± 2.38, and 4.00 ± 0.00 pg/mL 6, 12, and 18 months later (overall *p* = 0.006). In boys, the testosterone level was 0.83 ± 1.05 ng/mL at CPP diagnosis, 0.57 ± 0.85 ng/mL at formulation change, and 0.06 ± 0.03 ng/mL six months later (overall *p* = 0.04).

### 3.3 Changes in the clinical parameters

During the treatment period, Tanner pubertal staging of our subjects remained similar to their stage at diagnosis. The mean Tanner stage before treatment was 2.26 ± 0.59 (2.27 ± 0.52 for girls, 2.22 ± 0.83 for boys). The baseline breast development before treatment was at Tanner stage 2 in 75.8% of the patients, stage 3 in 21.2%, and stage 4 in 3.00%. The breast or pubic hair Tanner stage did not change during treatment in any of the girls, and no vaginal bleeding was observed. In boys, the average testicular volume was well maintained below 4 cc through treatment.

**Figure 2:**
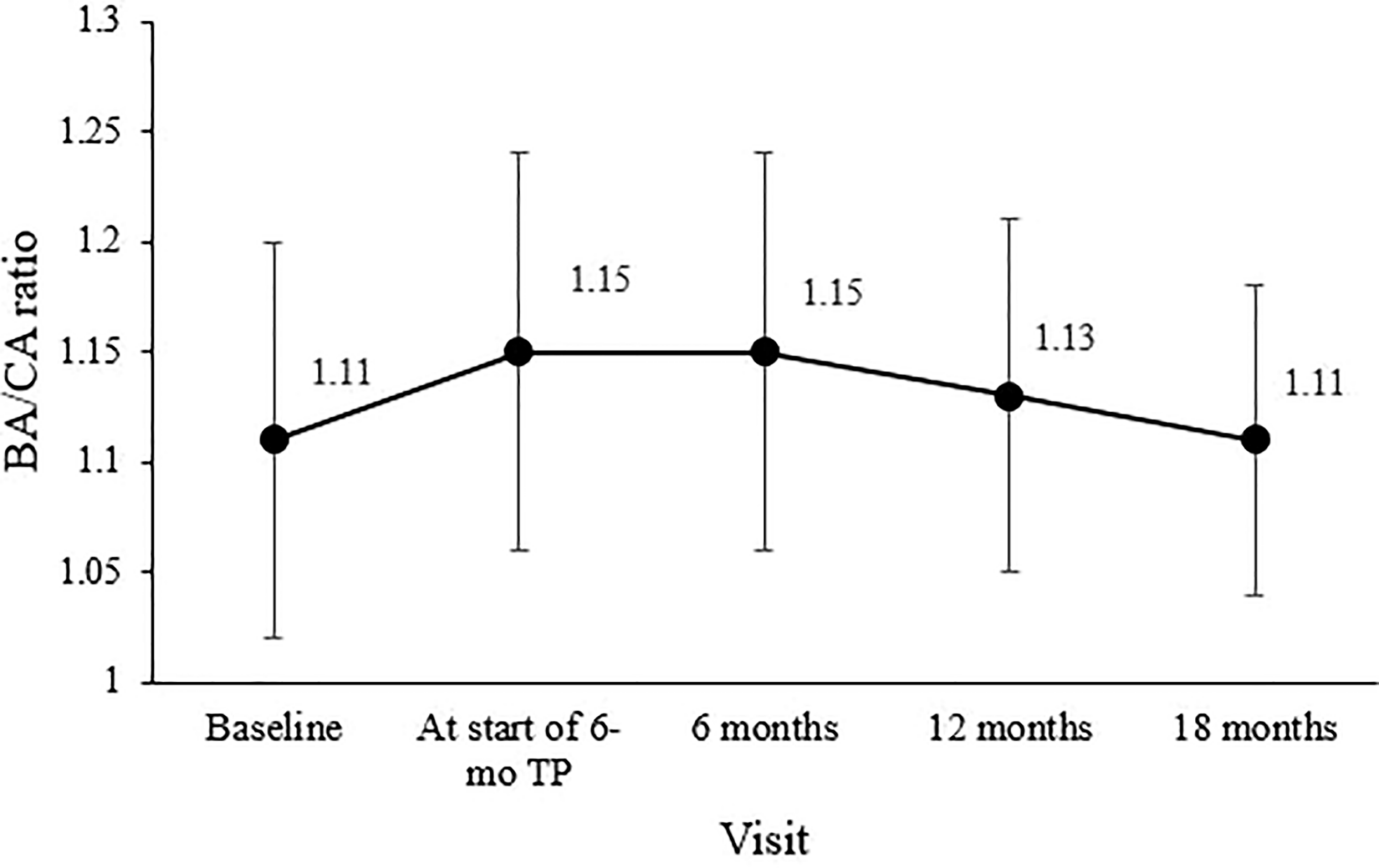
Changes in the mean bone age/chronological age (BA/CA) ratio during treatment. The overall significance was p = 0.007.

The bone age/chronological age ratio was 1.11 ± 0.09 at diagnosis and 1.15 ± 0.09 at the start of the 6-mo TP treatment. During the 18-month 6-mo TP treatment, the bone age/chronological age ratio decreased to 1.11 ± 0.08 (overall *p* = 0.007; **Figure 2**).

**Figure 3:**
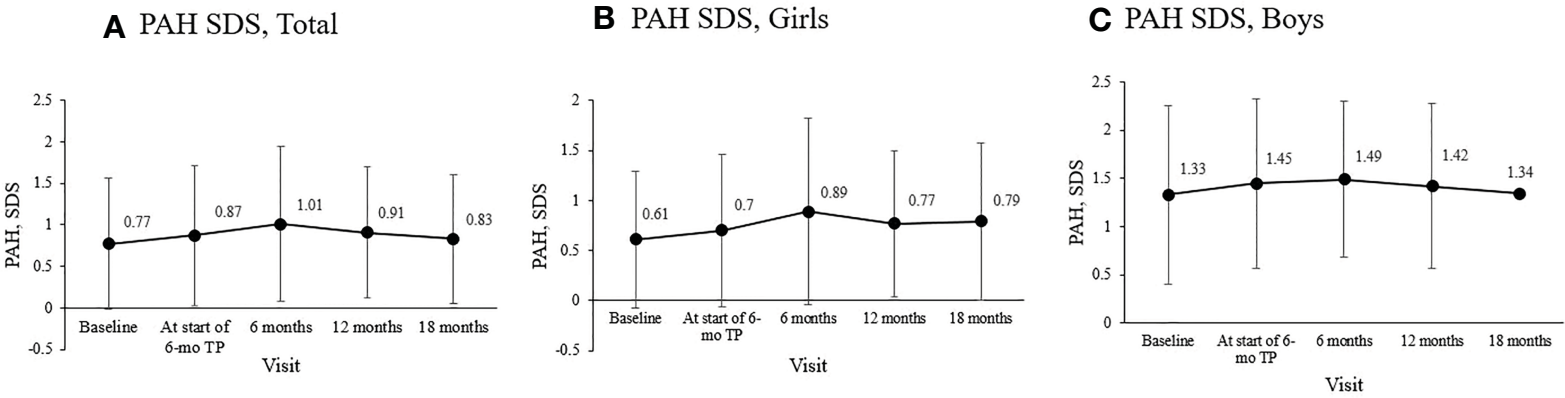
Changes in the mean PAH SDS during treatment. **(A)** The entire cohort. **(B)** Girls. **(C)** Boys. Predicted adult height, PAH; standard deviation score, SDS; TP, triptorelin pamoate.

Mean PAH SDS for the entire cohort was 0.77 ± 0.79 at diagnosis, 0.87 ± 0.84 before the 6-mo TP treatment initiation, and 1.01 ± 0.93 six months later. Mean PAH SDS remained above the initial SDS values throughout the treatment period (overall *p* = 0.038; **Figure 3A**). In girls, the mean PAH SDS was 0.61 ± 0.68 at diagnosis, 0.70 ± 0.76 at the formulation change, and 0.89 ± 0.93 six months later. Their PAH values at 6, 12, and 18 months of treatment with 6-mo TP were all higher than at diagnosis (overall *p* = 0.004; **Figure 3B**). In boys, although non-significant probably owing to the small sample number, the mean PAH SDS values after 6, 12, and 18 months of treatment with 6-mo TP were numerically higher than at the time of diagnosis (1.49 ± 0.81, 1.42 ± 0.86, and 1.34 ± 0.00, respectively; overall *p* = 0.387; **Figure 3C**).

### 3.4 Adverse events

Temporary induration and pain at the injection site were noted during the treatment period, but no long-lasting local complications occurred. Other complications, such as headache, rash, gastrointestinal symptoms, and menopausal symptoms, did not occur in any of the patients during the study period.

## 4 Discussion

This study analyzed the treatment effects of a 6-mo TP (22.5 mg triptorelin) formulation in children with CPP in various aspects, focusing on changes in hormones, bone age, and PAH. The 6-mo TP formulation recently approved for CPP offers greater convenience to children than other formulations, by reducing the injection frequency; however, related research is very limited (12). The 1-month dose presentation is still the most used, and usage of the 3-month dosage form is increasing (32–34); however, there is a lack of clinical experience worldwide regarding the use of the 6-month formulation (35). It has not yet been universalized, and doubts about its efficacy and safety persist among guardians and pediatric endocrinologists (1, 35). Therefore, the 6-mo TP formulation was not used from treatment initiation in any of the cases in this study. The change to 6-mo TP was mainly made in patients previously treated with the 1-month or 3-month formulation. The shift to the 6-month dosage form in this study was made at an average of four months from starting the GnRH agonist treatment.

To our knowledge, this was the second study, following the study by Klein et al. (35) from 2016, to investigate the efficacy and safety of 6-mo TP in children with CPP. Unlike this previous study (35), ours was a long-term study (>18 months), and PAH changes during treatment were analyzed. The previous study also used a simple leuprolide stimulation test with a single 30-min post-stimulation LH sample, which has been supported in several studies (36, 37). In contrast, we used the 90-min multi-sample gonadorelin stimulation test recommended as the reference standard test for CPP in other studies (38, 39). Of course, a global consensus on GnRH stimulation test and cutoff level for the diagnosis of CPP has not yet been established.

**Figure 4:**
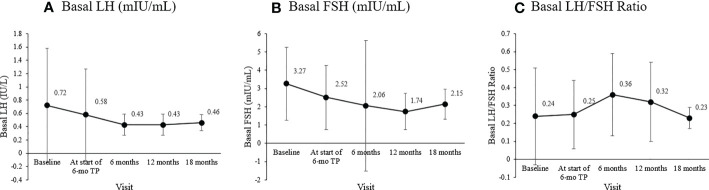
Changes in the mean basal gonadotropin levels during treatment. **(A)** Basal luteinizing hormone (LH; mIU/mL; overall p = 0.109). **(B)** Basal follicle-stimulating hormone (FSH; mIU/mL; overall p = 0.049). **(C)** Basal LH/FSH ratio (overall p = 0.098).

The primary outcome measure, the basal LH has been shown by many to be suitable for monitoring the effects of GnRH agonist treatment, and basal LH <0.6 IU/L was suggested as a cutoff for adequate suppression in CPP (22, 40). Throughout treatment, the basal LH level was well suppressed to below this cutoff value, suggesting that gonadotropic axis suppression was effectively achieved (**Figure 4A**) (20–22, 41), the basal FSH level was maintained at a prepubertal level (<2.5 IU/L; **Figure 4B**) (23), and the LH/FSH ratio was maintained below the 0.66 cutoff for nonprogressive precocious puberty (**Figure 4C**) (24, 25, 42).

Changes in serum estradiol in girls and testosterone in boys from baseline to our study’s end were consistent with gonadal suppression to prepubertal levels. The sex hormones cutoff values to differentiate prepubertal from pubertal state vary among laboratories. This study considered estradiol above 12.3 pg/mL and testosterone above 0.481 ng/mL as pubertal levels based on laboratory cutoffs and previous findings (5, 23). The baseline estradiol level at diagnosis might be at the prepubertal level in 40% of girls with CPP, as seen in our study. However, a significant decrease in basal estradiol during treatment indicates satisfactory hormonal control (1, 3, 43). Estradiol decreased from 7.35 pg/mL when we started using the 6-month formulation to an average of 4 pg/mL during the 18 months of treatment, well below the prepubertal level (**Figure 1A**). CPP can probably be ruled out if the baseline testosterone in boys is at the prepubertal level (26). The mean basal testosterone in our study exceeded the pubertal level but decreased significantly to prepubertal levels after the 6-mo TP was administered, and remained so for over a year (**Figure 1B**).

Secondary outcome measures were suppression of sex hormones, slowing the progression in the development of secondary characteristics and bone age maturation and increasing PAH. Clinical inhibition of sexual development during treatment was evaluated by periodic assessment of the Tanner stage. Suppression of bone age progression was determined based on a significant decrease in the bone age/chronological age ratio (3), which was maintained in this study at an average of 1.1 throughout treatment, similar to the pretreatment level. Furthermore, the bone age/chronological age ratio decreased significantly from 1.15 ± 0.09 at the 6-mo TP treatment initiation to 1.11 ± 0.07 18 months later, implying a gap between bone age and chronological age had been decreased due to suppression of bone age during treatment.

Another important end‐point considered was the PAH (44), calculated based on the height and bone age measured every six months after diagnosis. PAH and PAH SDS analysis indicated that PAH SDS increased along the treatment course. This was particularly noticeable in girls. This finding suggested that the final adult height will benefit from suppressing premature growth plate fusion by the 6-mo TP treatment.

A limitation of this study was that it was not a large-scale, long-term randomized comparative trial. The 6-mo TP was considered for patients meeting the inclusion criteria who were not short, overweight, or obese. Among these, the change to 6-mo TP was considered when patients had difficulties visiting the hospital because of the COVID-19 pandemic, resided in provincial regions or overseas, had severe injection phobia, or had an allergy to the 1- or 3-month injection form that required taking antihistamines. However, most of these were excluded from the study because they received 6-mo TP only once. Some could not afford the 6-month formulation because of their private medical insurance issue. Therefore, the number of patients fulfilling the inclusion criteria was limited. Future randomized comparative studies will need to overcome these limitations and compare treatment progress with the 1- or 3-month formulation to that of using the 6-month formulation starting at diagnosis. It is also worth noting that our patients’ PAH was rather high. All of our patients met the national reimbursement criteria for CPP treatment and had PAH less than TH at diagnosis. However, there is a possibility that there may have been cases in which the decision to start treatment should have been made with more follow-up. In addition, since the Bayley–Pinneau method for measuring PAH was developed in 1952 for Western children, there are parts that do not fit realistically, and adult height may have been overestimated (45).

Despite these limitations, this study analyzed various aspects of the long-term results of 6-mo TP administration in patients with CPP, including clinical, hormonal, and PAH changes. In addition, the study consistency was high because the same specialist performed its uniform treatment and examination at a single institution. Many follow-up studies are expected based on the positive effects of the 6-mo TP injection seen in this study. It is also expected that the CPP treatment paradigm will shift to the long-acting formulation.

## 5 Conclusion

This study confirmed the hypothalamic-pituitary-gonadal axis inhibitory effect of 6-mo TP. Furthermore, we confirmed that sexual development did not advance, bone age progression slowed, and the PAH SDS increased during treatment. This study provides extensive and practical information for pediatric endocrinologists by sharing our long-term clinical experience with 6-mo TP, which has not yet been widely studied.

## Data availability statement

The raw data supporting the conclusions of this article will be made available by the authors, without undue reservation.

## Ethics statement

The studies involving human participants were reviewed and approved by The Institutional Review Board of the Kangbuk Samsung Hospital. Written informed consent from the participants’ legal guardian/next of kin was not required to participate in this study in accordance with the national legislation and the institutional requirements.

## Author contributions

EY collected the data and drafted the manuscript. SK performed the statistical analysis and data interpretation. HJ, JYS, JWS, and DK collected and cleared the data. JK and EK contributed to the study design and takes responsibility for the integrity of the data and the accuracy of the data analysis. AY conceived and supervised the study, interpreted the results, and drafted the manuscript. All authors contributed to the article and approved the submitted version.

## References

[1] Carel JC Eugster EA Rogol A Ghizzoni L Palmert MR Antoniazzi F. Consensus statement on the use of gonadotropin-releasing hormone analogs in children. Pediatrics (2009) 123(4):e752-62. doi: 10.1542/peds.2008-1783 19332438

[2] Alikasifoglu A Vuralli D Gonc EN Ozon A Kandemir N. Changing etiological trends in Male precocious puberty: Evaluation of 100 cases with central precocious puberty over the last decade. Hormone Res Paediatrics (2015) 83(5):340–4. doi: 10.1159/000377678 25791832

[3] Carel JC Léger J. Clinical practice. precocious puberty. N Engl J Med (2008) 358(22):2366–77. doi: 10.1056/NEJMcp0800459 18509122

[4] Antoniazzi F Zamboni G. Central precocious puberty: current treatment options. Paediatr Drugs (2004) 6(4):211–31. doi: 10.2165/00148581-200406040-00002 15339200

[5] Latronico AC Brito VN Carel JC. Causes, diagnosis, and treatment of central precocious puberty. Lancet Diabetes Endocrinol (2016) 4(3):265–74. doi: 10.1016/S2213-8587(15)00380-0 26852255

[6] de Vries L Horev G Schwartz M Phillip M. Ultrasonographic and clinical parameters for early differentiation between precocious puberty and premature thelarche. Eur J Endocrinol (2006) 154(6):891–8. doi: 10.1530/eje.1.02151 16728550

[7] Zhu SY Du ML Huang TT. An analysis of predictive factors for the conversion from premature thelarche into complete central precocious puberty. J Pediatr Endocrinol Metab (2008) 21(6):533–8. doi: 10.1515/jpem-2008-210607 18717239

[8] Franzini IA Yamamoto FM Bolfi F Antonini SR Nunes-Nogueira VS. GnRH analog is ineffective in increasing adult height in girls with puberty onset after 7 years of age: a systematic review and meta-analysis. Eur J Endocrinol (2018) 179(6):381–90. doi: 10.1530/EJE-18-0473 30324797

[9] Lahlou N Carel JC Chaussain JL Roger M. Pharmacokinetics and pharmacodynamics of GnRH agonists: Clinical implications in pediatrics. J Pediatr Endocrinol Metab (2000) 13(Suppl 1):723–37. doi: 10.1515/JPEM.2000.13.S1.723 10969915

[10] Carel JC Lahlou N Guazzarotti L Joubert-Collin M Roger M Colle M. Treatment of central precocious puberty with depot leuprorelin. French leuprorelin trial group. Eur J Endocrinol (1995) 132(6):699–704. doi: 10.1530/eje.0.1320699 7788009

[11] Bertelloni S Mucaria C Baroncelli GI Peroni D. Triptorelin depot for the treatment of children 2 years and older with central precocious puberty. Expert Rev Clin Pharmacol (2018) 11(7):659–67. doi: 10.1080/17512433.2018.1494569 29957076

[12] Bangalore Krishna K Fuqua JS Rogol AD Klein KO Popovic J Houk CP. Use of gonadotropin-releasing hormone analogs in children: Update by an international consortium. Horm Res Paediatr (2019) 91(6):357–72. doi: 10.1159/000501336 31319416

[13] Zhang Y Ni J Zhang L Yu T Li X Xue P. The prevalence of precocious puberty among children in qufu city, Shandong province, China, a population-based study. Front Endocrinol (2022) 13. doi: 10.3389/fendo.2022.910119

[14] Kim YJ Kwon A Jung MK Kim KE Suh J Chae HW. Incidence and prevalence of central precocious puberty in Korea: An epidemiologic study based on a national database. J Pediatr (2019) 208:221–8. doi: 10.1016/j.jpeds.2018.12.022 30857777

[15] Kim SH Huh K Won S Lee KW Park MJ. A significant increase in the incidence of central precocious puberty among Korean girls from 2004 to 2010. PloS One (2015) 10(11):e0141844. doi: 10.1371/journal.pone.0141844 26539988 PMC4634943

[16] Cheuiche AV da Silveira LG de Paula LCP Lucena IRS Silveiro SP. Diagnosis and management of precocious sexual maturation: an updated review. Eur J Pediatr (2021) 180(10):3073–87. doi: 10.1007/s00431-021-04022-1 33745030

[17] Cao R Liu J Fu P Zhou Y Li Z Liu P. The diagnostic utility of the basal luteinizing hormone level and single 60-minute post GnRH agonist stimulation test for idiopathic central precocious puberty in girls. Front Endocrinol (Lausanne) (2021) 12:713880. doi: 10.3389/fendo.2021.713880 34456870 PMC8387794

[18] Neely EK Hintz RL Wilson DM Lee PA Gautier T Argente J. Normal ranges for immunochemiluminometric gonadotropin assays. J Pediatr (1995) 127(1):40–6. doi: 10.1016/S0022-3476(95)70254-7 7608809

[19] Ying Y Tang J Chen W Cai Z Niu WT. GnRH agonist treatment for idiopathic central precocious puberty can improve final adult height in Chinese girls. Oncotarget (2017) 8(65):109061–7. doi: 10.18632/oncotarget.22568 PMC5752503 29312590

[20] Durá-Travé T Gallinas-Victoriano F Malumbres-Chacon M Ahmed-Mohamed L Guindulain MJC Berrade-Zubiri S. Clinical data and basal gonadotropins in the diagnosis of central precocious puberty in girls. Endocr Connect (2021) 10(2):164–70. doi: 10.1530/EC-20-0651 PMC7983482 33416514

[21] Liang JT. Value of basal serum gonadotropin levels in the diagnosis of precocious puberty in girls. Zhongguo Dang Dai Er Ke Za Zhi (2012) 14(12):942–5. 23234783

[22] Lee PA Luce M Bacher P. Monitoring treatment of central precocious puberty using basal luteinizing hormone levels and practical considerations for dosing with a 3-month leuprolide acetate formulation. J Pediatr Endocrinol Metab (2016) 29(11):1249–57. doi: 10.1515/jpem-2016-0026 27740929

[23] Soldin OP Hoffman EG Waring MA Soldin SJ. Pediatric reference intervals for FSH, LH, estradiol, T3, free T3, cortisol, and growth hormone on the DPC IMMULITE 1000. Clin Chim Acta (2005) 355(1-2):205–10. doi: 10.1016/j.cccn.2005.01.006 PMC3636986 15820497

[24] Wang ZF Li GJ. Value evaluation of follicle stimulating hormone and luteinizing hormone in the diagnosis of precocious puberty in girls by ROC curve analysis. Zhongguo Dang Dai Er Ke Za Zhi (2012) 14(6):441–4. 22738452

[25] Supornsilchai V Hiranrat P Wacharasindhu S Srivuthana S Aroonparkmongkol S. Basal luteinizing hormone/follicle stimulating hormone ratio in diagnosis of central precocious puberty. J Med Assoc Thai (2003) 86(Suppl 2):S145–51. 12929982

[26] Partsch CJ Heger S Sippell WG. Management and outcome of central precocious puberty. Clin Endocrinol (Oxf) (2002) 56(2):129–48. doi: 10.1046/j.0300-0664.2001.01490.x 11874402

[27] Kim JH Yun S Hwang SS Shim JO Chae HW Lee YJ. The 2017 Korean national growth charts for children and adolescents: Development, improvement, and prospects. Korean J Pediatr (2018) 61(5):135–49. doi: 10.3345/kjp.2018.61.5.135 PMC5976563 29853938

[28] Bayer LM. *Radiographic atlas of skeletal development of the hand and wrist: Second edition.* 1959. Calif Med (1959) 91(1):53.

[29] Sorva R Tolppanen EM Lankinen S Perheentupa J. Growth evaluation: parent and child specific height standards. Arch Dis Child (1989) 64(10):1483–7. doi: 10.1136/adc.64.10.1483 PMC1792769 2817934

[30] Bayley N Pinneau SR. Tables for predicting adult height from skeletal age: revised for use with the greulich-pyle hand standards. J Pediatr (1952) 40(4):423–41. doi: 10.1016/S0022-3476(52)80205-7 14918032

[31] De Sanctis V Soliman AT Di Maio S Soliman N Elsedfy H. Long-term effects and significant adverse drug reactions (ADRs) associated with the use of gonadotropin-releasing hormone analogs (GnRHa) for central precocious puberty: A brief review of literature. Acta BioMed (2019) 90(3):345–59. doi: 10.23750/abm.v90i3.8736 PMC7233750 31580327

[32] Fuld K Chi C Neely EK. A randomized trial of 1- and 3-month depot leuprolide doses in the treatment of central precocious puberty. J Pediatr (2011) 159(6):982–7.e1. doi: 10.1016/j.jpeds.2011.05.036 21798557

[33] Carel JC Blumberg J Seymour C Adamsbaum C Lahlou N. Three-month sustained-release triptorelin (11.25 mg) in the treatment of central precocious puberty. Eur J Endocrinol (2006) 154(1):119–24. doi: 10.1530/eje.1.02056 16382000

[34] Chung LY Kang E Nam HK Rhie YJ Lee KH. Efficacy of triptorelin 3-month depot compared to 1-month depot for the treatment of Korean girls with central precocious puberty in single tertiary center. J Korean Med Sci (2021) 36(34):e219. doi: 10.3346/jkms.2021.36.e219 34463062 PMC8405405

[35] Klein K Yang J Aisenberg J Wright N Kaplowitz P Lahlou N. Efficacy and safety of triptorelin 6-month formulation in patients with central precocious puberty. J Pediatr Endocrinol Metab (2016) 29(11):1241–8. doi: 10.1515/jpem-2015-0376 26887034

[36] Lawson ML Cohen NM. Use of a rapid subcutaneous LHRH stimulation test for assessing the effectiveness of LHRH agonist therapy in children with central precocious puberty. Pediatr Res (1998) 43(4):79–9. doi: 10.1203/00006450-199804001-00471

[37] Lawson ML Cohen N. A single sample subcutaneous luteinizing hormone (LH)-releasing hormone (LHRH) stimulation test for monitoring LH suppression in children with central precocious puberty receiving LHRH agonists. J Clin Endocrinol Metab (1999) 84(12):4536–40. doi: 10.1210/jcem.84.12.6181 10599714

[38] Vukovic R Milenkovic T Soldatovic I Pekic S Mitrovic K Todorovic S. Triptorelin stimulated luteinizing hormone concentrations for diagnosing central precocious puberty: study of diagnostic accuracy. Endocrine (2022) 75(3):934–41. doi: 10.1007/s12020-021-02947-z PMC8616750 34826116

[39] Rodanaki M Rask E Lodefalk M. A randomized trial of the effect of a GnRH analogue injection on ghrelin levels in girls. Horm Res Paediatr (2022) 95(5):442–51. doi: 10.1159/000526147 35896083

[40] Schubert S Hvelplund AH Handberg A Hagstroem S Leunbach TL. Elevated pre-injection basal luteinizing hormone concentrations are common in girls treated for central precocious puberty. J Clin Res Pediatr Endocrinol (2021) 13(2):204–11. doi: 10.4274/jcrpe.galenos.2020.2020.0210 PMC8186339 33374097

[41] Lee HS Yoon JS Park KJ Hwang JS. Increased final adult height by gonadotropin-releasing hormone agonist in girls with idiopathic central precocious puberty. PloS One (2018) 13(8):e0201906. doi: 10.1371/journal.pone.0201906 30133462 PMC6104939

[42] Oerter KE Uriarte MM Rose SR Barnes KM Cutler GB. Gonadotropin secretory dynamics during puberty in normal girls and boys. J Clin Endocrinol Metab (1990) 71(5):1251–8. doi: 10.1210/jcem-71-5-1251 2121771

[43] Brito VN Latronico AC Arnhold IJ Mendonça BB. Update on the etiology, diagnosis and therapeutic management of sexual precocity. Arq Bras Endocrinol Metabol (2008) 52(1):18–31. doi: 10.1590/S0004-27302008000100005 18345393

[44] Carel JC Lahlou N Roger M Chaussain JL. Precocious puberty and statural growth. Hum Reprod Update (2004) 10(2):135–47. doi: 10.1093/humupd/dmh012 15073143

[45] Brito VN Latronico AC Cukier P Teles MG Silveira LF Arnhold IJ. Factors determining normal adult height in girls with gonadotropin-dependent precocious puberty treated with depot gonadotropin-releasing hormone analogs. J Clin Endocrinol Metab (2008) 93(7):2662–9. doi: 10.1210/jc.2007-2183 18460564

